# Kidney biopsy-based epidemiologic analysis shows growing biopsy rate among the elderly

**DOI:** 10.1038/s41598-021-04274-9

**Published:** 2021-12-29

**Authors:** Adél Molnár, Mbuotidem Jeremiah Thomas, Attila Fintha, Magdolna Kardos, Deján Dobi, András Tislér, Nóra Ledó

**Affiliations:** 1 Department of Internal Medicine and Oncology, Faculty of Medicine, Semmelweis University, Korányi Sándor utca 2/a, Budapest, 1083 Hungary; 2 Institute of Translational Medicine, Semmelweis University, Budapest, Hungary; 3 2nd Department of Pathology, Faculty of Medicine, Semmelweis University, Budapest, Hungary

**Keywords:** Glomerular diseases, Vasculitis syndromes

## Abstract

Systematic registration and examination of biopsy-related data in Central and Eastern Europe are scarce, while the health condition of the population is worse compared to other more developed countries. We aim to create a database and analyze the distribution and temporal variation of the renal biopsy diagnoses in Hungary, including the effect of the recent coronavirus pandemic. The diagnoses were standardized according to the recommendation of the European Renal Association. Native biopsy samples processed between January 1, 2006, and December 31, 2020, were analyzed. During the 15 years, 2140 native kidney biopsies were performed. The number of samples increased from 24.5 to 57.9 per million person-years and the median age from 37 to 51 years (*p* < 0.0001). The predominance of glomerular diseases was stable. The most frequent glomerulopathy was IgA nephropathy (21.5%), followed by focal segmental glomerulosclerosis (17.7%), and membranous nephropathy (15.7%). Trends showed the rise of ANCA-associated vasculitis. During the coronavirus pandemic, there was a decrease in the number of kidney biopsies and the proportion of membranous nephropathies. The diagnostic trends in our database showed increasing biopsy rates among the elderly and the growing frequencies of age-related diseases, which emphasizes the importance of altering medical focus according to demographic changes in this area.

## Introduction

Percutaneous kidney biopsy is a valuable method in diagnosing certain kidney diseases^1^. Analysis of the biopsy specimens provides information on the pathogenesis, disease activity and facilitates therapeutic decisions.

Current epidemiologic trends on the incidence and prevalence of renal diseases are available throughout Western Europe collected in large national or regional registries^2–7^. There are some registries published from Central Europe^8,9^, however, there has not been any study published in the English literature about the incidence and prevalence of renal biopsies in Hungary in the last decades.

Our study aimed to assess the temporal trends of the incidence and changing patterns of kidney biopsies based on clinicopathological data in Hungary. The main reason for assuming differences in the renal biopsy-related data in this region compared to Western European countries is the higher rate of diabetes mellitus, malignant and cardiovascular diseases^10,11^. These factors convey the risk of developing certain kidney-related injuries^12–14^ that may become evident by analyzing kidney biopsy data.

Our second objective was to evaluate biopsy rates and diagnoses in 2020 compared to the previous years. The presence of the global coronavirus pandemic may have had an impact on the availability of various diagnostic processes, such as kidney biopsies. The distribution of renal diseases diagnosed by biopsies during the pandemic may give us valuable information on the crucial indications of this diagnostic tool.

## Results

### Demographics

Between 2006 and 2020, 2140 native and 111 transplant biopsies were evaluated. The latter ones were excluded from our analysis. The total number of native biopsy diagnoses was 2296.

The male/female ratio was 49.8%/50.2%, with a mean age of 44.2 ± 21.9 years (Table 1). The youngest patient was 4 months, while the oldest was 90 years old. The median age was 46 years. 18.3% of the recorded biopsies were obtained from children, 61.3% from adults, and 20.4% from the elderly. The median age increased significantly (*p* < 0.0001) in the last 6 years (2015–2020), and the proportion of the patients above 18 years increased as well (*p* < 0.0001) (Table 1).

**Table 1:** Demographics.

Category	All	2006–2008	2009–2011	2012–2014	2015–2017	2018–2020	*p*
Age (years)	44.2 ± 21.9 (46; 0.25–90)	37.8 ± 22.7 (37; 0.8–89)	37.5 ± 23.2 (38; 0.25–81)	38.7 ± 22.4 (40; 3–80)	47.5 ± 20.5 (49; 0.75–88)	49.0 ± 19.9 (51; 2–90)	< 0.0001
Gender (m/f)	1065(49.8)/1075(50.2)	144(49.0)/150(51.0)	145(49.2)/150(50.8)	146(53.9)/125(46.1)	304(49.0)/316(51.0)	326(49.4)/334(50.6)	NS
Children (≤ 18 y)	391 (18.3)	81 (27.6)	92 (31.2)	74 (27.3)	73 (11.8)	71 (10.8)	< 0.0001
Adult (19–65 y)	1312 (61.3)	172 (58.5)	161 (54.6)	154 (56.8)	400 (64.5)	425 (64.4)	< 0.0001
Elderly (≥ 66 y)	437 (20.4)	41 (13.9)	42 (14.2)	43 (15.9)	147 (23.7)	164 (24.8)	< 0.0001
Biopsy rate (pmp)	36.3	24.5	24.2	22.3	55.2	57.9	

The age of the patients is presented as mean ± SD (median; range); the gender and the age group are presented as the patient number with the percentage (%).

*p* values show the result of the statistical analysis of the difference between the 3-year intervals.

Kruskal–Wallis test (age) or Chi-square test were used (gender, age groups), accordingly.

*NS* nonsignificant, *m* male, *f* female, *y* year, *pmp* per million person-years.

There were no significant changes in the gender distribution during this period. The biopsy rate increased in the last 6 years (2015–2020); the average rate was 36.3 per one million person-years in 15 years (Table 1). The average gender-based rate was 38.2 and 34.6 per one million person-years (males and females, respectively).

### Frequencies of the main disease groups

**Figure 1:**
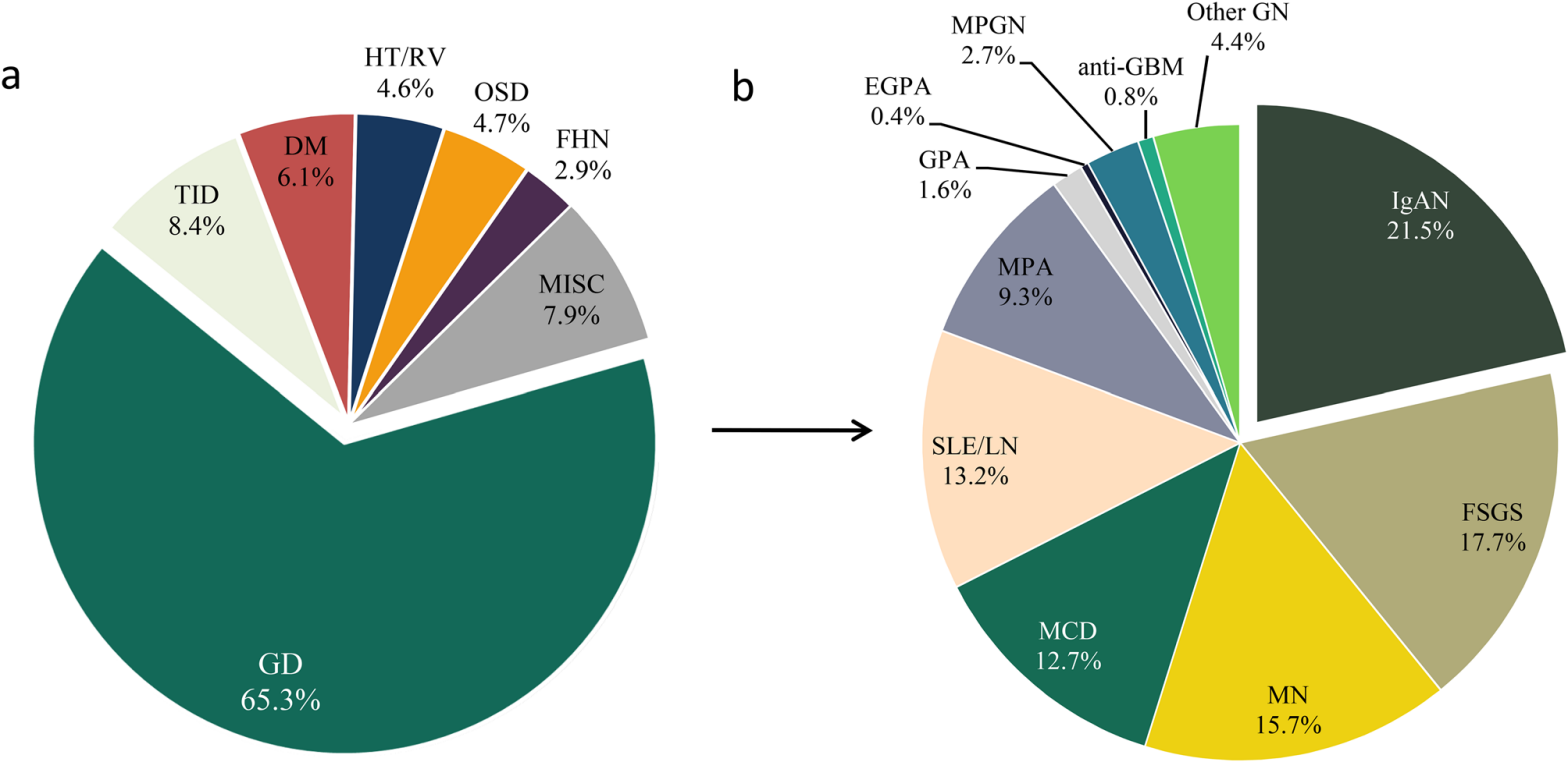
Frequencies of the main groups throughout 15 years (**a**). *GD* glomerular diseases, *TID* tubulointerstitial diseases, *DM* diabetes mellitus, *HT/RV* hypertension/renal vascular disease, *OSD* other systemic disease affecting the kidney, *FHN* familial/hereditary nephropathies, *MISC* miscellaneous diseases. Frequencies of glomerular diseases throughout the 15 years (**b**). *IgAN* IgA nephropathy—histologically proven and Henoch-Schönlein purpura/nephritis, *FSGS* focal segmental glomerulosclerosis, *MN* membranous nephropathy (primary and secondary), *MCD* minimal change disease, *SLE/LN* systemic lupus erythematosus/lupus nephritis, *MPA* microscopic polyangiitis, *GPA* granulomatosis with polyangiitis, *EGPA* eosinophilic granulomatosis with polyangiitis, *MPGN* membranoproliferative glomerulonephritis, *Other GN* other glomerulonephritis.

Overall, the biopsies had glomerular diseases (GD) with 65.3%, followed by tubulointerstitial diseases (TID, 8.4%), diabetes mellitus (DM, 6.1%), other systemic diseases (OSD, 4.7%), renal vascular diseases (HT/RV, 4.6%), familial/hereditary nephropathies (FHN, 2.9%), and miscellaneous (MISC, 7.9%) diagnoses. (Table 2, Fig. 1a).

**Table 2:** Frequency of the main renal diagnostic categories in the specified periods.

Category	All	2006–2008	2009–2011	2012–2014	2015–2017	2018–2020	*p*	p*
	n = 2296	n = 331	n = 327	n = 288	n = 678	n = 672		
GD	1499 (65.3)	205 (61.9)	204 (62.4)	189 (65.6)	445 (65.6)	456 (67.9)	NS	NS
TID	192 (8.4)	44 (13.3)	33 (10.1)	25 (8.7)	51 (7.5)	39 (5.8)	0.001	0.007
DM	141 (6.1)	15 (4.5)	17 (5.2)	18 (6.3)	44 (6.5)	47 (7.0)	NS	NS
HT/RV	106 (4.6)	17 (5.1)	16 (4.9)	5 (1.7)	48 (7.1)	20 (3.0)	0.001	0.009
OSD	109 (4.7)	5 (1.5)	16 (4.9)	17 (5.9)	31 (4.6)	40 (6.0)	NS	NS
FHN	67 (2.9)	18 (5.4)	15 (4.6)	14 (4.9)	8 (1.2)	12 (1.8)	< 0.0001	0.02
MISC	182 (7.9)	27 (8.2)	26 (8.0)	20 (6.9)	51 (7.5)	58 (8.6)	NS	NS

The table shows the frequencies of the main diagnostic groups.

They are presented as absolute numbers and percentages (%).

*p* values show the result of the statistical analysis of the difference between the 3-year intervals.

p* values demonstrate the difference between the last 3 years (2018–2020) and the first 12 years (2006–2017).

Chi-square test was used.

*GD* glomerular diseases, *TID* tubulointerstitial diseases, *DM* diabetes mellitus, *HT/RV* hypertension/renal vascular disease, *OSD* other systemic disease affecting the kidney, *FHN* familial/hereditary nephropathies, *MISC* miscellaneous diseases, *NS* nonsignificant.

The group of glomerular diseases encompassed a wide range of diagnoses of different etiology, which we analyzed separately, and present the findings in this article later.

The group of tubulointerstitial disease (n = 192) comprised primarily of drug-induced tubulointerstitial nephritis (n = 176, 91.7%), but we encountered also autoimmune mechanism (n = 7, 3.6%), calcium (n = 4, 2.1%) and uric acid deposition diseases (n = 2, 1%), and HIV nephropathy (n = 3, 1.6%). The group of other systemic diseases (n = 109) comprised of amyloidosis (n = 90, 82.6%) and thrombotic microangiopathy (n = 19, 17.4%). In the familial/hereditary nephropathy group (n = 67), we found Alport-syndrome (n = 13, 19.4%), thin basement membrane disease (n = 46, 68.7%), nephronophthisis (n = 2, 3%), primary hyperoxaluria (n = 4, 6%), and genetically proven congenital thrombotic microangiopathy (n = 2, 3%). The miscellaneous group (n = 182) assembled chronic kidney failure of unknown etiology (n = 48, 26.4%), acute pyelonephritis (n = 5, 2.7%), acute kidney injury (n = 32, 17.6%), tumors (n = 5, 2.7%), oligomeganephronia (n = 1, 0.5%), *sine morbo* diagnoses (n = 8, 4.4%), or specimens that did not have diagnosis due to technical problems (n = 83, 45.6%).

### 3-year interval trends show dominance of glomerular diseases

For evaluation of the changing patterns over time, we analyzed the trends between 3-year intervals (Table 2).

Glomerular diseases were the most frequent throughout the years without any change in the fraction of total distribution.

TID decreased in the last three years (2018–2020) (*p* = 0.007), as well as FHN (*p* = 0.02), and HT/RV (*p* = 0.009). The decrease of the FHN diagnoses was strongly affected by the increasing age, while the decrease in HT/RV was independent of the increasing age of the patients. (Suppl. Table 1).

Even though we demonstrated a steady increase in the frequency of DM, it was not statistically significant (*p* = 0.745).

### Gender analysis in the main diagnostic categories; males prevail in diabetes mellitus

**Figure 2:**
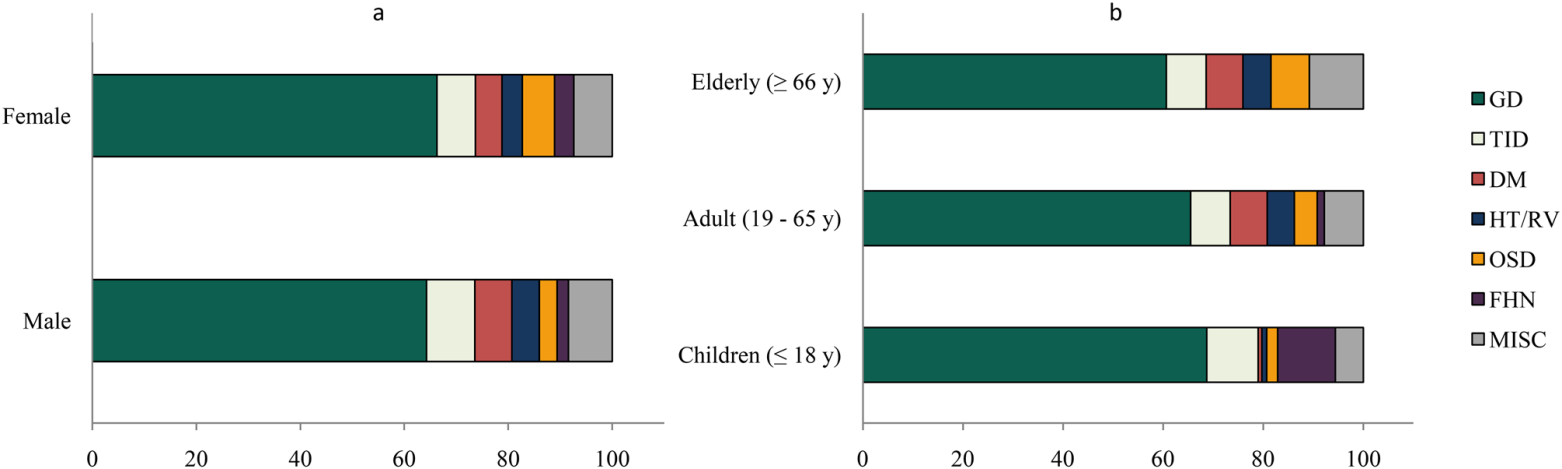
The fraction of total of the main diagnoses according to gender (**a**) and age groups (**b**). They are presented percentages (%). *GD* glomerular diseases, *TID* tubulointerstitial diseases, *DM* diabetes mellitus, *HT/RV* hypertension/renal vascular disease, *OSD* other systemic disease affecting the kidney, *FHN* familial/hereditary nephropathies, *MISC* miscellaneous diseases, *y* years.

Among the distribution of the diagnoses, gender plays a significant role (*p* = 0.0004). Glomerular diseases displayed equal distribution between genders. We observed a significantly higher number of males in DM (*p* = 0.025) and significantly more females in the OSD (*p* = 0.002) and the FHN group (*p* = 0.038) (Table 3, Fig. 2a).

**Table 3:** Percentages of the main renal diagnostic categories according to gender and age.

Category	2006–2020						
	Male n = 1161	Female n = 1135	*p*	< 18 y n = 412	19–65 y n = 1416	> 66 y n = 468	*p*
GD (%)	64.3	66.3	NS	68.4	65.8	60.9	NS
TID (%)	9.3	7.4	NS	10.7	7.8	7.9	NS
DM (%)	7.1	5.1	0.025	0.7	7.3	7.3	< 0.0001
HT/RV (%)	5.3	3.9	NS	1.0	5.4	5.6	< 0.0001
OSD (%)	3.4	6.2	0.002	2.2	4.5	7.7	0.001
FHN (%)	2.2	3.7	0.038	11.4	1.4	0.0	< 0.0001
MISC (%)	8.4	7.4	NS	5.6	7.7	10.7	0.012

The table shows the fraction of total of the main diagnoses according to gender and age.

They are presented as percentages (%).

*p* values show the result of the statistical analysis of the difference between gender or the age groups.

Chi-square test was used.

*GD* glomerular diseases, *TID* tubulointerstitial diseases, *DM* diabetes mellitus, *HT/RV* hypertension/renal vascular disease, *OSD* other systemic disease affecting the kidney, *FHN* familial/hereditary nephropathies, *MISC* miscellaneous diseases, *NS* nonsignificant, *y* years.

### Age analysis in the main diagnostic categories

Regarding the age groups, we observed a significant difference in the distribution of the diagnosis between children, adults, and the elderly (*p* < 0.0001).

Glomerular diseases were dominant across all age groups (60.9–68.4%).

We noticed a higher number of diabetes mellitus (*p* < 0.0001), hypertension/renal vascular (*p* < 0.0001), other systemic diseases (*p* = 0.001), and miscellaneous diagnoses (*p* = 0.012) in the adult and elderly group. As expected, familial/hereditary nephropathies were diagnosed more excessively in children (*p* < 0.0001) (Table 3, Fig. 2b).

### Main diagnostic categories, genders within the age groups

We also adjusted the genders within the age groups over the years.

Examination showed female dominance in the other systemic disease group in adults (*p* = 0.0045) and male dominance in the elderly diabetes mellitus group (*p* = 0.0312) (Suppl. Table 2, Suppl. Fig. 1).

### Frequency of glomerular diseases; IgAN is the most frequent glomerulopathy in our database

Since the majority of the specimens were glomerular diseases, we conducted further analysis on this group.

The IgA nephropathy group includes IgA nephropathy and IgA vasculitis (Henoch-Schönlein purpura). The membranous nephropathy group contains both primary and secondary forms. Other glomerulonephritis group collects entities that occurred infrequently in our database: IgM nephropathy (n = 3), diffuse endocapillary glomerulonephritis (n = 41), histologically indeterminate glomerulonephritis (n = 15), cryoglobulinemia (n = 6), and C1q nephropathy (n = 1).

ANCA-associated vasculitis group consisted of microscopic polyangiitis (MPA), granulomatosis with polyangiitis (GPA), and eosinophil granulomatosis with polyangiitis (EGPA).

The most frequent glomerulopathy was IgA nephropathy (IgAN, 21.5%), followed by focal segmental glomerulosclerosis (FSGS, 17.7%), membranous nephropathy (MN, 15.7%), minimal change disease (MCD, 12.7%), systemic lupus erythematosus with lupus nephritis (SLE/LN, 13.2%), ANCA-associated vasculitis (11.3%, with the diagnosis of MPA in 9.3%), other glomerulonephritis group (4.4%), membranoproliferative glomerulonephritis (MPGN, 2.7%) and anti-GBM nephropathy (0.8%). (Table 4, Fig. 1b).

**Table 4:** Frequencies of glomerular diseases reported in the specified periods.

Category	All	2006–2008	2009–2011	2012–2014	2015–2017	2018–2020	*p*	p*
	n = 1499	n = 205	n = 204	n = 189	n = 445	n = 456		
IgAN	322 (21.5)	48 (23.4)	47 (23.0)	42 (22.2)	84 (18.9)	101 (22.1)	NS	NS
FSGS	265 (17.7)	44 (21.5)	41 (20.1)	38 (20.1)	77 (17.3)	65 (14.3)	NS	0.027
MN	235 (15.7)	32 (15.6)	25 (12.3)	26 (13.8)	77 (17.3)	75 (16.4)	NS	NS
MCD	190 (12.7)	30 (14.6)	35 (17.2)	24 (12.7)	60 (13.5)	41 (9.0)	0.038	0.004
SLE/LN	198 (13.2)	21 (10.2)	25 (12.3)	25 (13.2)	67 (15.1)	60 (13.2)	NS	NS
MPA	140 (9.3)	12 (5.9)	14 (6.9)	16 (8.5)	37 (8.3)	61 (13.4)	0.012	< 0.001
GPA	24 (1.6)	2 (1.0)	5 (2.5)	3 (1.6)	4 (0.9)	10 (2.2)	NS	NS
EGPA	6 (0.4)	-	-	1 (0.5)	2 (0.4)	3 (0.7)	NS	NS
MPGN	41 (2.7)	2 (1.0)	6 (2.9)	4 (2.1)	10 (2.2)	19 (4.2)	NS	0.021
Anti-GBM	12 (0.8)	2 (1.0)	1 (0.5)	2 (1.1)	3 (0.7)	4 (0.9)	NS	NS
Other GN	66 (4.4)	12 (5.9)	5 (2.5)	8 (4.2)	24 (5.4)	17 (3.7)	NS	NS

The table shows the frequencies of glomerular diseases.

They are presented as absolute numbers and percentages (%).

*p* values show the result of the statistical analysis of the difference between the 3-year intervals.

p* values demonstrate the difference between the last 3 years (2018–2020) and the first 12 years (2006–2017).

Chi-square test was used.

*IgAN* IgA nephropathy—histologically proven and Henoch-Schönlein purpura/nephritis, *FSGS* focal segmental glomerulosclerosis, *MN* membranous nephropathy (primary and secondary), *MCD* minimal change disease, *SLE/LN* systemic lupus erythematosus/lupus nephritis, *MPA* microscopic polyangiitis, *GPA* granulomatosis with polyangiitis, *EGPA* eosinophilic granulomatosis with polyangiitis, *MPGN* membranoproliferative glomerulonephritis, *Other GN* other glomerulonephritis, *NS* nonsignificant.

### 3-year trends in glomerular diseases indicate a rise of ANCA-associated vasculitis and MPGN

IgAN remained the prevailing diagnosis over the years.

During the 15-years, we observed a decrease in the biopsy diagnoses of MCD (*p* = 0.038). FSGS also decreased in the last three years (2018–2020) (*p* = 0.027), however, these changes were age-dependent.

ANCA-associated vasculitis showed an increasing frequency in the 15-year period (*p* = 0.004). Within this group, MPA demonstrated the same trend (*p* = 0.012), while changes for GPA and EGPA were not significant. The increase in MPA diagnoses was partly explained by the increasing age of the patients.

An increase was found in MPGN in the last three years (2018–2020) (*p* = 0.021), which was age and gender independent (Table 4, Suppl. Table 1).

### Glomerular diseases and gender: male dominance in MN

The leading diagnoses of males were IgA nephropathy (30.6%), while lupus nephritis proved to be the most frequent glomerulonephritis (21.8%) in females.

Analyzing the subgroups, we found a significant male dominance in MN (*p* = 0.022) and IgAN (*p* < 0.0001), and female dominance in MPA (*p* < 0.0001) and SLE/LN (*p* < 0.0001).

**Figure 3:**
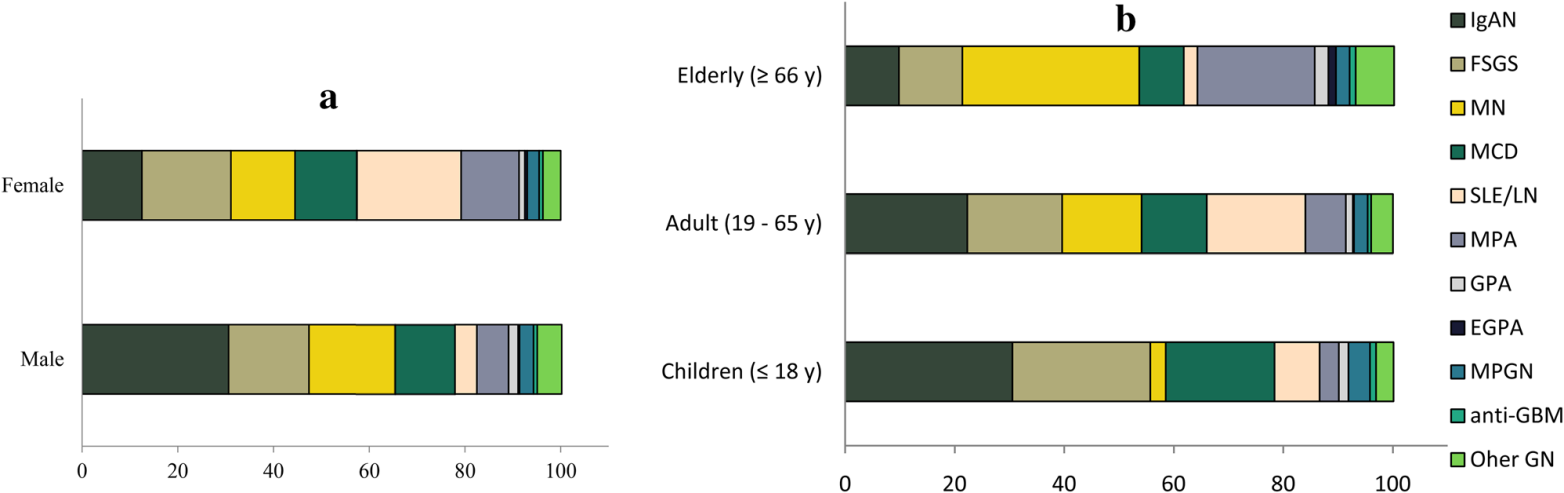
The fraction of total of the glomerular diseases according to gender (**a**) and age groups (**b**). They are presented as percentages (%). *IgAN* IgA nephropathy—histologically proven and Henoch-Schönlein purpura/nephritis, *FSGS* focal segmental glomerulosclerosis, *MN* membranous nephropathy (primary and secondary), *MCD* minimal change disease, *SLE/LN* systemic lupus erythematosus/lupus nephritis, *MPA* microscopic polyangiitis, *GPA* granulomatosis with polyangiitis, *EGPA* eosinophilic granulomatosis with polyangiitis, *MPGN* membranoproliferative glomerulonephritis, *Other GN* other glomerulonephritis, *y* years.

The distribution of MCD, MPG, and anti-GBM was divided between the genders almost equally (Table 5, Fig. 3a).

**Table 5:** Percentages of the glomerular diseases according to gender and age.

Category	2006–2020						
	Male n = 746	Female n = 753	*p*	< 18 y n = 282	19–65 y n = 932	> 66 y n = 285	*p*
IgAN (%)	30.6	12.5	< 0.0001	30.5	22.3	9.8	< 0.0001
FSGS (%)	16.8	18.6	NS	25.2	17.3	11.6	0.001
MN (%)	18.0	13.4	0.022	2.8	14.5	32.3	< 0.0001
MCD (%)	12.5	12.9	NS	19.9	11.9	8.1	< 0.0001
SLE/LN (%)	4.6	21.8	< 0.0001	8.2	18.0	2.5	< 0.0001
MPA (%)	6.6	12.1	< 0.0001	3.5	7.4	21.4	< 0.0001
GPA (%)	2.0	1.2	NS	1.8	1.3	2.5	NS
EGPA (%)	0.3	0.5	NS	–	0.2	1.4	0.017
MPGN (%)	2.9	2.5	NS	3.9	2.5	2.5	NS
Anti-GBM (%)	0.8	0.8	NS	1.1	0.6	1.1	NS
Other GN (%)	5.1	3.7	NS	3.2	4.0	7.0	NS

The table shows the fraction of total of the glomerular diseases according to gender and age.

They are presented as percentages (%).

*p* values show the result of the statistical analysis of the difference between the genders or age groups.

Chi-square test was used.

*IgAN* IgA nephropathy—histologically proven and Henoch-Schönlein purpura/nephritis, *FSGS* focal segmental glomerulosclerosis, *MN* membranous nephropathy (primary and secondary), *MCD* minimal change disease, *SLE/LN* systemic lupus erythematosus/lupus nephritis, *MPA* microscopic polyangiitis, *GPA* granulomatosis with polyangiitis, *EGPA* eosinophilic granulomatosis with polyangiitis, *MPGN* membranoproliferative glomerulonephritis, *Other GN* other glomerulonephritis, *NS* nonsignificant, *y* years.

### Glomerular diseases and age groups: MN dominate in the elderly

IgAN was the most frequent glomerulonephritis in the children and adults, while MN topped the elderly (32.3%) group.

Within the age groups, we noticed an adult/elderly dominance in MN (*p* < 0.0001), MPA (*p* < 0.001), and in EGPA (*p* = 0.017).

On the contrary, SLE/LN demonstrated children/adult dominance (*p* < 0.0001).

MCD (*p* < 0.0001), FSGS (*p* = 0.001), and IgAN (*p* < 0.0001) proved to be the most prevalent in children.

Although MPGN was higher in children, and there were more GPA in the elderly, these differences were not significant (Table 5, Fig. 3b).

### Glomerular diseases; genders within age groups

Furthermore, we broke down the glomerular diagnoses based on gender within the age groups.

A significant male dominance in IgAN in each age groups (*p* < 0.0001 children, *p* < 0.0001 adults, *p* = 0.015 elderly) and a female predominance in SLE/LN (*p* < 0.0001 children, *p* < 0.0001 adults, *p* = 0.018 elderly) were observed. Additionally, we observed a female predominance in children’s group in MPA (*p* = 0.019), and male dominance in MN (*p* = 0.001) among adults (Suppl. Table 3, Suppl. Fig. 2).

### Additional findings and rare diseases

Further analysis demonstrated a female dominance in amyloidosis (*p* = 0.026), especially in the AA (Amyloid A) amyloidosis group (*p* = 0.008).

During the years, we discovered a significant change in the distribution of light chain deposition disease (LCDD, *p* < 0.0001). LCDD increased significantly in the last 3-year period, which was partly explained by the increasing age (Suppl. Table 1).

During these 15 years, we encountered some rarities: three IgM nephropathies, one C1q nephropathy, three HIV nephropathies, and five immunotactoid glomerulopathies.

### Coronavirus pandemic affected the frequencies of kidney biopsies and the histopathologic diagnoses

**Figure 4:**
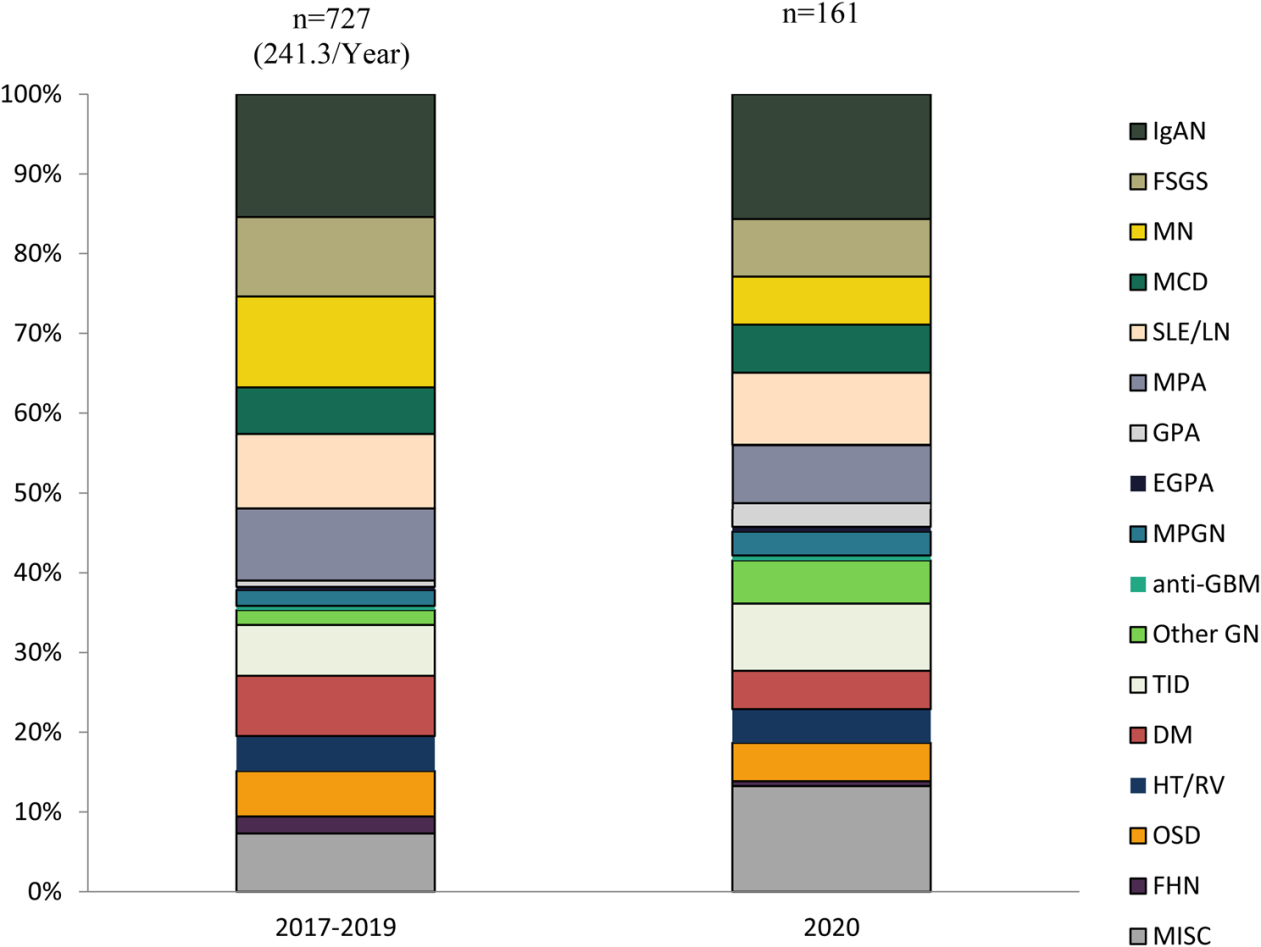
Coronavirus pandemic affected the frequencies of kidney biopsies and the histopathologic diagnoses. *IgAN* IgA nephropathy—histologically proven and Henoch-Schönlein purpura/nephritis, *FSGS* focal segmental glomerulosclerosis, *MN* membranous nephropathy (primary and secondary), *MCD* minimal change disease, *SLE/LN* systemic lupus erythematosus/lupus nephritis, *MPA* microscopic polyangiitis, *GPA* granulomatosis with polyangiitis, *EGPA* eosinophilic granulomatosis with polyangiitis, *MPGN* membranoproliferative glomerulonephritis, *Other GN* other glomerulonephritis. *GD* glomerular diseases, *TID* tubulointerstitial diseases, *DM* diabetes mellitus, *HT/RV* hypertension/renal vascular disease, *OSD* other systemic disease affecting the kidney, *FHN* familial/hereditary nephropathies, *MISC* miscellaneous diseases.

In 2020, there was a decrease in the number of kidney biopsies compared to the average of the previous three years (2017–2019): 161 biopsies, 43.4 per one million person-years vs. 242.3 biopsies per year, 64.2 per one million person-year between 2017 and 2019. Among the biopsy diagnoses, we found a decrease in membranous nephropathy (10 in 161 (6.2%) in 2020 vs. 86 in 727 (11.8%) between 2017 and 2019, *p* = 0.038), an increase in GPA (6 in 727 (0.8%) between 2017 and 2019 vs. 5 in 161 (3.1%) in 2020, *p* = 0.018), other GN (14 in 727 (1.9%) between 2017 and 2019 vs. 9 in 161 (5.6%) in 2020, *p* = 0.008), and miscellaneous diseases (55 in 727 (7.6% between 2017 and 2019 vs. 22 in 161 (13.7%) in 2020, *p* = 0.013) (Fig. 4).

## Discussion

Our 15-year retrospective study of renal biopsies provides comprehensive data about demographics and the prevalence of kidney diseases in Hungary. Although our report was conducted in a single pathological center, it covers almost half of the Hungarian population and can therefore be generalized.

Our database has a slight female dominance, which might root in the fact that the male/female demographic ratio decreases with the increase of age^15^. However, the gender-based biopsy rate showed a relatively higher number of males who have gone under renal biopsy. This compares well with other European studies^1^.

The mean age was 44.2 ± 21.9 years, which coincides with similar reports in other registries so far^1^. The increase in biopsy samples from 2014 was partly due to the addition of other tertiary nephrology centers to the catchment area but the contribution of the increasingly aging population and subsequent higher biopsy rate in the elderly population cannot be disregarded either. This implies that there is a long-term outlook even for senior patients, and suggests an increasing life expectancy, giving an indirect hint of improving health care and social conditions^16^.

The average biopsy rate of 36.3 per one million person-years lags behind most of the European reports^17^, however, from 2015, our data collection rate has increased significantly and seems to be catching up. We assume many reasons behind the lower rates. First, the catchment area is scattered, and there are just a few hospitals that maintain a regular connection with the university pathological department. In addition, a conservative approach to biopsies may have caused the lower number of biopsy incidences. Financial considerations can also hold the biopsy rates back^1,9^. Nevertheless, biopsy rates in Hungary have significantly improved over the last few years. This may be due to several reasons. The decision to biopsy has been positively impacted by the increased proficiency and low complication rates of the procedure. This has resulted in increased ease of performing renal biopsy procedures by nephrologists and subsequently has led to improved biopsy skills. Additionally, nephrologists with higher expertise in performing renal biopsies, tend to have lower threshold for performing these procedures. Furthermore, nephrologists and associated professionals who feel more confident in their fellows’ competence are more likely to request more biopsies. The changing emphasis of performing a biopsy during medical training may also contribute to the increase in the biopsy rates. Training pattern has changed in the last decades, leading to a change in the nephrologist population. Many doctors who have trained abroad and those who have familiarized themselves with renal biopsy procedures during their training, become acquainted with it and are more likely to perform it. Thus, with time the biopsy rate could grow exponentially^18^. The widespread availability of the internet, medical search engines, and journals provide better availability of high-quality, up-to-date information on kidney diseases, and the utility of the biopsy, which also has contributed to the lowering threshold for renal biopsy. Nonetheless, we cannot exclude the real increase in the incidence of kidney diseases as a contributing factor to the increased renal biopsy rates as well as the aging population. With aging, certain kidney diseases and renal manifestations of systemic diseases may occur as they have more time to develop.

**Figure 5:**
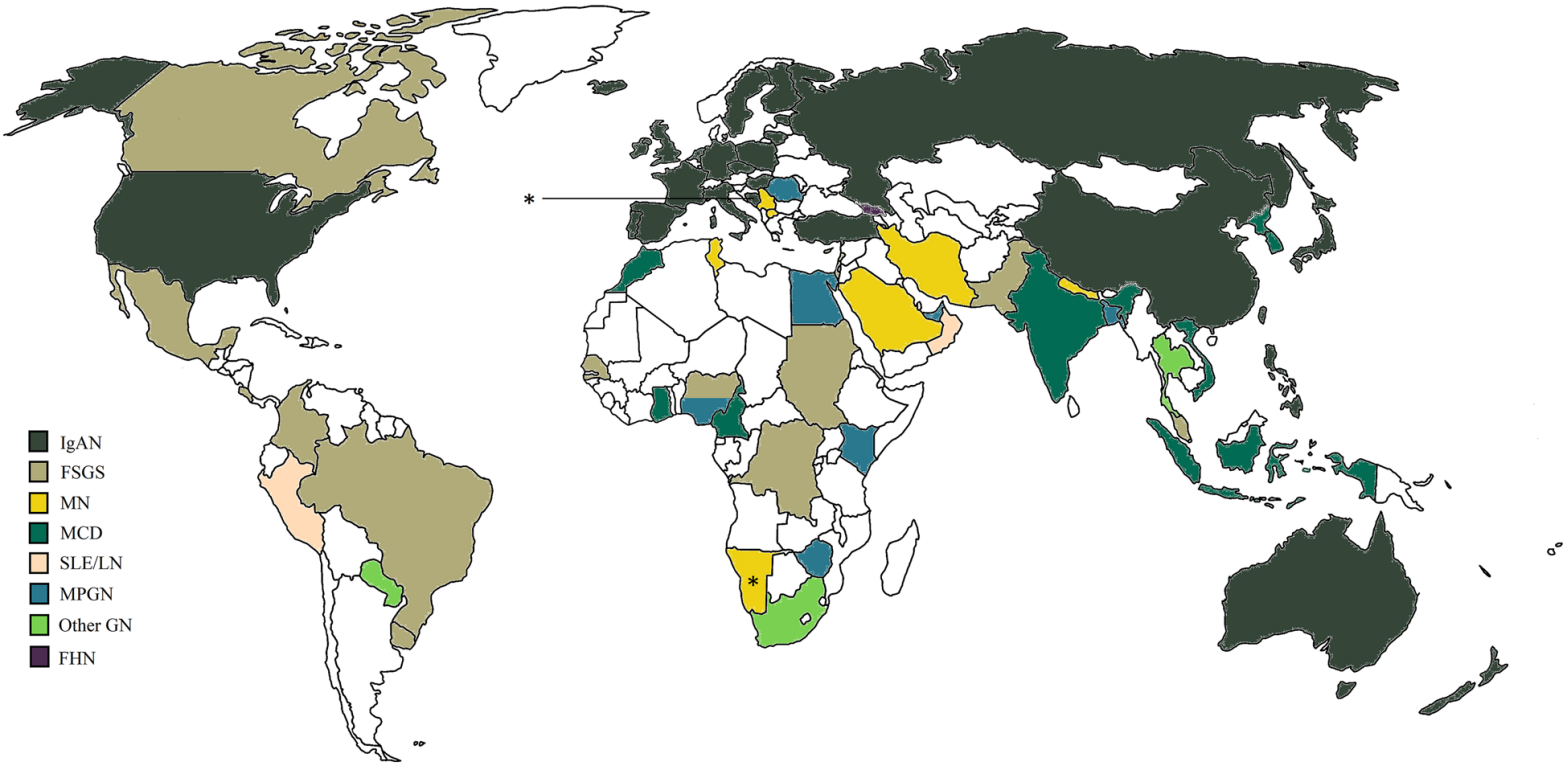
Overview of the most frequent renal diseases in renal registries and studies around the world. Biopsy indication was heterogenous except in Cameroon, Senegal, Ghana, and Zaire, where only nephrotic syndrome was considered. Biopsies were performed on adults ± children, except Namibia, where only children were enrolled in the study. Information was not found from countries left white. This Figure was created with Paint software (Microsoft Windows 10 v20H2). The world map template was downloaded from https://www.dreamstime.com/royalty-free-stock-images-empty-world-map-image4506299 under Royalty-free license, Dreamstime LLC (Brentwood, TN, US). *IgAN* IgA nephropathy—histologically proven and Henoch-Schönlein purpura/nephritis, *FSGS* focal segmental glomerulosclerosis, *MN* membranous nephropathy (primary and secondary), *MCD* minimal change disease, *SLE/LN* systemic lupus erythematosus/lupus nephritis, *MPGN* membranoproliferative glomerulonephritis, *Other GN* other glomerulonephritis, *FHN* familial/hereditary nephropathies, *MISC* miscellaneous diseases. *Only children were examined.

The results confirmed that glomerular diseases prevail over the other diagnoses similar to most of the registries^3,8,17,19–43^. Within glomerular diseases, IgA nephropathy was the most common entity, which correlates well with another Hungarian registry in a different region^44,45^ and other registries in different countries^2–6,8,9,19–21,29–31,46–58^. Interestingly, there were some countries, mainly outside of Europe, where membranous nephropathy^22,23,26,38,59,60^ or FSGS/MCD were the most frequently diagnosed^24,25,32–35,37,40,43,61–70^. Membranoproliferative glomerulonephritis prevailed in Africa and mostly in Eastern countries^7,27,28,39,43,71–74^. In some countries, lupus nephritis, diffuse endocapillary glomerulonephritis, IgM nephropathy and familial/hereditary nephropathies dominated the renal biopsies^36,41,75–78^ (Fig. 5). The discrepancy of the most frequent diagnoses indicates not only a different genetic, lifestyle and environmental background but also raises awareness for the heterogeneity of the biopsy indications^17^.

Sine morbo diagnoses are mostly believed to be produced by the inherent problem of the sampling errors, resulting in false-negative samples, however, true negative cases cannot be ruled out either.

The key predictor of biopsy adequacy is the number of glomeruli, which changes depending on the kind of glomerular disease. In general, missing the impaired glomeruli in the sample is 10% if the bioptic sample includes 10 glomeruli and it drops to 1% if the sample contains 20 glomeruli. As a result, for an acceptable sample, at least 10 glomeruli are required^79^. This notion is supported by the recent guideline of the Kidney Disease: Improving Global Outcomes (KDIGO) organization on the management of glomerular diseases^80^. In case it is not yielded during the sampling, chances of missing the afflicted part of the kidney increase.

In our study, negative cases made up for 0.38% of the total biopsies. This rate varies between 0.3 and 8.5% in the international reports^9,22,36,47,48,57,61,70^. A biopsy is still the “gold standard” for the diagnostic evaluation of glomerular diseases and the biopsy should be performed when the value of the information obtained from the biopsy exceeds the risk entailed^80^. Most of the renal biopsy indications (e.g. nephrotic syndrome, acute nephritic syndrome, affected kidney in systemic diseases, etc.) rule out the true negative results, hence these are characteristic for kidney diseases. This can explain the small rate of true negative samples compared to other diagnostic tests. Indications for renal biopsy in the countries where the report on negative samples was available, were generally similar. However, the number of the negative samples increased in direct proportion to the rate of biopsies. This correlation draws attention to the threshold for the execution of a renal biopsy: countries that perform a renal biopsy with more subtle clinical or laboratory abnormalities have a higher biopsy rate and increased number of negative histological results, while countries with low negative sample results and biopsy rate may interpret the indications rather strictly. In addition, local resources are also likely to determine the prevailing practice on performing kidney biopsies^80^.

The prevalence of hypertension increases with age^81^, and renal manifestation may be parallel this tendency. In this study, we found less hypertension/renovascular disease-related diagnosis in the biopsy trends despite the increasing age. This might indicate better medical support and blood pressure control for the patients, even in the elderly. On the other hand, we cannot exclude the dilution effect entirely: the rise of the biopsy rate may have contributed to a relatively lower number of hypertensive/renovascular main diagnosis. Here we must note that many specimens had arterial hyalinosis as a sign of hypertension which was not described as the main diagnosis if it was an indirect result or secondary effect. The hypertension/renal vascular main category represents the specimens if the signs of hypertension were explicit or no other pathognomic lesion was found.

The increase of age was accompanied by a surplus of microscopic polyangiitis, and a lower number of familial diseases. As indicated in other studies, the average age is over 50 years in MPA, indicating a higher prevalence in older patients^82^. The increasing number of LCDD and MPGN in the biopsy trends might be attributed to monoclonal gammopathies, which also rise with the aging population. MPGN, as a heterogeneous range of disorders, is often clinically under-recognized and hence, under-diagnosed^83^. Although monoclonal gammopathies may have a diverse and even distinct morphology, the most common pattern is MPGN^84–86^.

The number of membranous nephropathies showed increase with age and demonstrated a male dominance. This may be explained by the gradually aging population^10^ and the corresponding increase in the incidence of malignancies^87^. This finding correlates well with international studies^88^. Nevertheless, the increasing level of air pollutants cannot be excluded either^89^. We demonstrated a slightly higher proportion of MN cases than in the neighboring countries, which may be attributed to our worldwide number one ranking in lung and colorectal cancers and a superior place in ovarian and bladder cancers^90^. We also proved that MN occurs at a younger age in males which may be attributed to their lower participation in screening tests, worse diet, and lifestyle differences^91^. It is also worth noting that the availability of anti-PLA2R (phospholipase-A-2-receptor) titer measurements widened our diagnostic arsenal, and in some situations may lead to a decrease in the biopsy incidence in primary MN cases^92^.

Younger patients were burdened more by lupus nephritis, IgA nephropathy, minimal change disease, and focal segmental glomerulosclerosis. The relative early manifestation of these diseases suggests the possibility of genetic involvement.

The prevalence of diabetic nephropathy varied in the reviewed articles. Its overall prevalence in this study was higher than in most countries^17^. It is worth noting that the population in Hungary has one of the highest overweight and obesity rates in Europe^15^. On the other hand, a Western German study^3^ showed a 3.6-fold higher rate of diabetic nephropathy in their study. The discrepancies suggest a variance in the indications of biopsy. The relatively low incidence of diabetic nephropathy in our study compared to theirs may be due to the fact that performing a renal biopsy in diabetes mellitus is necessary only when an unexpectedly high rate of proteinuria or renal function decline is present. However, early stages of diabetic nephropathy may present with advanced structural damage despite the fairly normal kidney function^93^. This suggests that approaching biopsy indications with a less restrictive attitude may be advantageous and draws attention to the poor secondary prevention measures in Hungary.

MPA is known to have a rather even gender distribution^94^. According to some reports, it has a slight male predominance^95–97^. However, gross comparison in our study showed a significant female dominance. This cannot be explained only by the shrinking number of males with age, since we observed female dominance in all age groups. It raises attention to the possibility, that although MPA is equal and even a bit more frequent in males, the renal manifestation may be affected by gender, and may occur more often in females.

The female dominance of amyloidosis is associated with the female dominance of AA amyloidosis. Most of the patients with this diagnosis had an underlying rheumatoid arthritis, which is more frequent in females. The rest was associated with inflammatory bowel diseases. AA amyloidosis also reflects the prevalence of chronic inflammatory conditions over time.

Furthermore, in this study, the incidence of ANCA-associated glomerulonephritis increased over time. This trend correlates well with previous reports in other epidemiologic studies^98–100^. ANCA-associated vasculitis is more prevalent in the older population^101^, therefore the aging population could explain its increasing incidence. The increased incidence may also be attributed to an actual increase in incidence, change in classification criteria, and wider availability of diagnostic ANCA serology tests^102,103^. Moreover, increased recognition may be due to increased awareness by the clinicians because of a more thorough education^98^.

Due to the COVID-19 pandemic, the healthcare system had a major challenge worldwide. Accordingly, in 2020 the renal biopsy rate was lower, compared to the previous years. Many Hungarian nephrology departments had to switch to urgent care for patients with COVID-19 from March to May and November to December in 2020, which reduced the number of biopsies that otherwise could have been performed. In addition, patients with less severe symptoms and stable kidney disease avoided hospital visits due to fear of the coronavirus. We hypothesized that during the pandemic only the portion of kidney diseases with rapid progression or severe symptoms, and for which treatment needed a histopathological confirmation ended up in biopsies. Of course, serology results with these patients were non-contributory. This underlines the diagnostic value and indication for biopsies even in these difficult times. Although our database did not have large case numbers in 2020, we found that the rate of MN decreased significantly. In the primary forms of MN where the anti-PLA2R is present, renal biopsies could be disregarded, especially in a pandemic situation. The rise of GPA can be explained partly by the better awareness of the disease, but the effect of the pandemic cannot be excluded either. More data are needed in the future to explain this trend of the disease.

Many countries run renal biopsy registries to have a more comprehensive insight into epidemiologic data, to improve research possibilities^5,9,20,24,47,49–51,55,61,104^. These registries also contain clinical data which can enhance a more in-depth understanding of the diseases. Recently the Hungarian Society of Nephrology has also established a renal biopsy registry, which will aid to conduct clinicopathological research. To this end with this article, we would also like to take the initiative to develop a high-functioning database and encourage both clinicians and pathologists to take a share of filling an international biopsy registry, which would be very important quality feedback to our overall clinical work.

Our retrospective study presents the long-term trends in kidney diseases diagnosed by renal biopsy in Hungary. The diagnostic trends in our database showed increasing biopsy rates among the elderly and the growing frequencies of age-related diseases. Trends of 2020 showed that the availability of kidney biopsies reduced during the COVID-19 pandemic, but renal biopsy remained an important diagnostic tool even in difficult times.

The establishment of not only national but international kidney biopsy registries should be encouraged and supported by scientific societies, as it is very important to compare these trends internationally, which can help to improve the quality control of nephrology care worldwide.

## Methods

### Demographics of kidney biopsy samples

We analyzed renal biopsy specimens retrospectively that were examined at the 2nd Department of Pathology, Semmelweis University, between January 2006 and December 2020, a period of 15 years.

Samples arrived from 28 different secondary and tertiary nephrology departments from Northern and Central Hungary from four Hungarian counties, including the capital, Budapest. Centers included both adult and pediatric care facilities.

Population estimates were retrieved from the Hungarian Central Statistical Office. The average population in Hungary between 2006 and 2020 was 9,916,101 persons, while the average population of the catchment area during this time period was 3,932,556 persons. Consequently, the mean background population in the examined area provides 39.7% of the population in Hungary.

The mean population density in these areas in this period was 107 inhabitants/km^2^. This is 1.2 times more than the mean population density of Hungary and 3.19 times more than the European average in this period.

### Histological assessment

According to our protocol, all specimens were stained with the same techniques and evaluated systematically by light microscopy, immunofluorescent, and electron microscope. Paraffin-embedded kidney tissue sections for light microscopy were routinely stained with hematoxylin and eosin, periodic-acid Schiff, Masson’s trichrome, Congo red, and Jones’ methenamine silver stains. For immunofluorescence examination, specimens were labelled with IgG, IgA, IgM, C3c, C4c, C1q, fibrin, kappa chain, and lambda chain conjugated fluorescent dye. If indicated for Alport syndrome, staining for collagen IV alpha 5 chain was also used. Between 2006 and 2019, specimens were assessed individually by two experienced nephropathologists (M.K., A.F.). In 2020, another experienced nephropathologist joined the assessments (D.D.). In most cases, clinical information provided by the attending clinicians aided their work.

All samples were included in our analysis, even sampling errors (e.g., adipose tissue). For the analysis, pathological findings documented in paper or electronic medical records were entered in a Microsoft Excel (version 2016) database. We registered the patients’ age, gender, primary, secondary and tertiary diagnosis (if more than one histological features were available), and the institution where the biopsy was performed. Repeated kidney biopsies were marked.

### Diagnoses

Terminologies were used as described in the European Renal Association-European Dialysis and Transplant Association (ERA-EDTA) coding system^105^. Based on this nomenclature, we divided the diagnoses into seven large renal diagnostic categories: glomerular diseases, tubulointerstitial diseases, diabetes mellitus, hypertension, other systemic diseases, familial nephropathies, and miscellaneous renal disorders.

Transplant kidney biopsies were excluded from the analysis.

In our tables, we present only those diagnostic categories of the ERA-EDTA coding system, where we had at least one diagnosis during the 15-year-period.

For better comparison with previous studies, we divided patients by age and sex. Patients 18 years or younger were considered as children, those between 19 and 65 years as adults, and those 66 years or older were considered as elderly.

We also grouped diagnoses in 3-year intervals and examined 2020 separately, to be able to assess the effect of the COVID-19 (coronavirus disease 2019) pandemic on biopsy rates.

### Statistical analysis

Data were stored in an Excel (Microsoft, version 2016) database file. Statistical analysis was performed using Excel, GraphPad (GraphPad Prism 9.0.0), and IBM SPSS Statistics 27 software programs. Chi-square and Fisher’s exact test were used to compare categorical variables and Kruskal–Wallis test for continuous variables based on the result of the Shapiro–Wilk normality test. We also conducted logistic regression analysis with binary dependent variables.

Categorical variables are expressed as number (percentage), continuous variables as mean ± standard deviation, and median with range. Two-tailed *p* values < 0.05 were considered statistically significant.

All analyses were performed in accordance with relevant guidelines and regulations and informed consent was obtained from all subjects and/or their legal guardian(s) for further analyses at the time point of the biopsies. The study was approved by the Semmelweis University Regional and Institutional Committee of Science and Research Ethics (SE RKEB 225/2018).

## Supplementary Information

Supplementary Information.
