# Supplementary material for: Kidney biopsy-based epidemiologic analysis shows growing biopsy rate among the elderly
Source: Sci Rep. 2021 Dec 29;11:24479. doi: 10.1038/s41598-021-04274-9 (PMC8716536; doi:10.1038/s41598-021-04274-9)
Supplement: Supplementary file 1 — Supplementary Information. [file 41598_2021_4274_MOESM1_ESM.pdf]

SUPPLEMENTAL MATERIAL

Kidney biopsy-based epidemiologic analysis shows growing biopsy rate among the elderly

Adél Molnár, Mbuotidem Jeremiah Thomas, Attila Fintha, Magdolna Kardos, Deján Dobi, András Tislér, Nóra Ledó

Suppl. Table 1: Logistic regression analysis of the frequency of diagnoses, where significant difference in the last period (2018-2020) was present; analysis of the effect of age and gender, as co-variants

Sig.: significance (p), Exp(B): odds ratio, C.I.: confidence interval

TID (Tubulointerstitial Disease) - Variables in the Equation

		B	S.E.	Wald	df	Sig.	Exp(B)	95% C.I.for EXP(B)	
								Lower	Upper
Step 1[a]	2018 - 2020(1)	-,516	,189	7,445	1	,006	,597	,412	,865
	Age in years	,002	,004	,417	1	,518	1,002	,995	1,009
	Gender(1)	,179	,157	1,292	1	,256	1,196	,879	1,628
	Constant	-2,458	,197	155,990	1	,000	,086		

a. Variable(s) entered on step 1: 2018 - 2020, Age in years, Gender.

HT/RV (Hypertension / Renovascular Disease) - Variables in the Equation

		B	S.E.	Wald	df	Sig.	Exp(B)	95% C.I.for EXP(B) Lower	Upper
Step 1[a]	2018 - 2020(1)	-,794	,256	9,632	1	,002	,452	,274	,746
	Age in years	,024	,005	20,939	1	,000	1,024	1,014	1,034
	Gender(1)	,366	,205	3,172	1	,075	1,442	,964	2,157
	Constant	-4,114	,313	172,915	1	,000	,016		

a. Variable(s) entered on step 1: 2018 - 2020, Age in years, Gender.

FHN (Familial / Hereditary Nephropathies) - Variables in the Equation

		B	S.E.	Wald	df	Sig.	Exp(B)	95% C.I.for EXP(B) Lower	Upper
Step 1[a]	2018 - 2020(1)	-,200	,335	,357	1	,550	,818	,424	1,579
	Age in years	-,066	,008	63,200	1	,000	,936	,921	,951
	Gender(1)	-,522	,264	3,899	1	,048	,594	,354	,996
	Constant	-1,153	,232	24,753	1	,000	,316		

a. Variable(s) entered on step 1: 2018 - 2020, Age in years, Gender.

FSGS (Focal Segmental Glomerulosclerosis) - Variables in the Equation

		B	S.E.	Wald	df	Sig.	Exp(B)	95% C.I.for EXP(B)	
								Lower	Upper
Step 1[a]	2018 - 2020(1)	-,238	,154	2,385	1	,123	,789	,583	1,066
	Age in years	-,013	,003	18,753	1	,000	,987	,981	,993
	Gender(1)	-,131	,133	,963	1	,326	,878	,676	1,139
	Constant	-1,294	,150	74,499	1	,000	,274		

a. Variable(s) entered on step 1: 2018 - 2020, Age in years, Gender.

MCD (Minimal Change Disease) - Variables in the Equation

		B	S.E.	Wald	df	Sig.	Exp(B)	95% C.I.for EXP(B)	
								Lower	Upper
Step 1[a]	2018 - 2020(1)	-,360	,186	3,730	1	,053	,698	,484	1,005
	Age in years	-,022	,004	37,720	1	,000	,978	,971	,985
	Gender(1)	-,019	,154	,015	1	,901	,981	,726	1,326
	Constant	-1,352	,165	67,177	1	,000	,259		

a. Variable(s) entered on step 1: 2018 - 2020, Age in years, Gender.

MPA (Microscopic polyangiitis) - Variables in the Equation

		B	S.E.	Wald	df	Sig.	Exp(B)	95% C.I.for EXP(B)	
								Lower	Upper
Step 1[a]	2018 - 2020(1)	,392	,182	4,638	1	,031	1,480	1,036	2,115
	Age in years	,040	,005	59,245	1	,000	1,041	1,030	1,052
	Gender(1)	-,670	,186	12,946	1	,000	,512	,355	,737
	Constant	-4,589	,325	198,852	1	,000	,010		

a. Variable(s) entered on step 1: 2018 - 2020, Age in years, Gender.

MPGN (Membranoproliferative Glomerulonephritis) - Variables in the Equation

		B	S.E.	Wald	df	Sig.	Exp(B)	95% C.I.for EXP(B)	
								Lower	Upper
Step 1[a]	2018 - 2020(1)	,770	,325	5,602	1	,018	2,159	1,141	4,085
	Age in years	-,006	,007	,737	1	,391	,994	,979	1,008
	Gender(1)	,116	,320	,131	1	,717	1,123	,600	2,103
	Constant	-4,042	,401	101,815	1	,000	,018		

a. Variable(s) entered on step 1: 2018 - 2020, Age in years, Gender.

LCDD (Light Chain Deposition Disease) - Variables in the Equation

		B	S.E.	Wald	df	Sig.	Exp(B)	95% C.I.for EXP(B)	
								Lower	Upper
Step 1[a]	2018 - 2020(1)	2,214	,637	12,086	1	,001	9,153	2,627	31,893
	Age in years	,061	,018	11,661	1	,001	1,063	1,026	1,100
	Gender(1)	,300	,484	,384	1	,536	1,349	,523	3,481
	Constant	-9,670	1,308	54,638	1	,000	,000		

a. Variable(s) entered on step 1: 2018 - 2020, Age in years, Gender.

Suppl. Table 2: Percentage of main groups according to genders within the age groups

Category	All (%)								
	≤ 18 y		*P*	19 – 65 y		*P*	≥ 66 y		*P*
	M	F		M	F		M	F	
GD (%)	68,6	68,3	*NS*	64,5	67,3	*NS*	59,3	62,3	*NS*
TID (%)	13,2	8,2	*NS*	9,2	6,4	*NS*	6,0	9,5	*NS*
DM (%)	0,5	1,0	*NS*	8,1	6,5	*NS*	10,2	4,8	*0.0312*
HT/RV (%)	1,0	1,0	*NS*	6,2	4,4	*NS*	6,5	4,8	*NS*
OSD (%)	1,5	2,9	*NS*	3,0	6,2	*0.0045*	6,5	8,7	*NS*
FHN (%)	8,3	14,4	*NS*	1,1	1,8	*NS*	-	-	*NS*
MISC (%)	6,9	4,3	*NS*	8,0	7,4	*NS*	11,6	9,9	*NS*
n	204	208	-	741	675	-	216	252	-

Suppl. Table 2. The table shows the fraction of total of main diagnoses according to genders within the age groups. They are presented as percentages (%). P values show the result of the statistical analysis of the difference between the genders within the age groups. Chi-square and Fisher's exact test were used. GD: glomerular diseases, TID: tubulointerstitial diseases, DM: diabetes mellitus, HT/RV: hypertension/renal vascular disease, OSD: other systemic disease affecting the kidney, FHN: familial/hereditary nephropathies, MISC: miscellaneous diseases, NS: nonsignificant, y: years.

Suppl. Figure 1: Percentage of main groups according to genders within the age groups

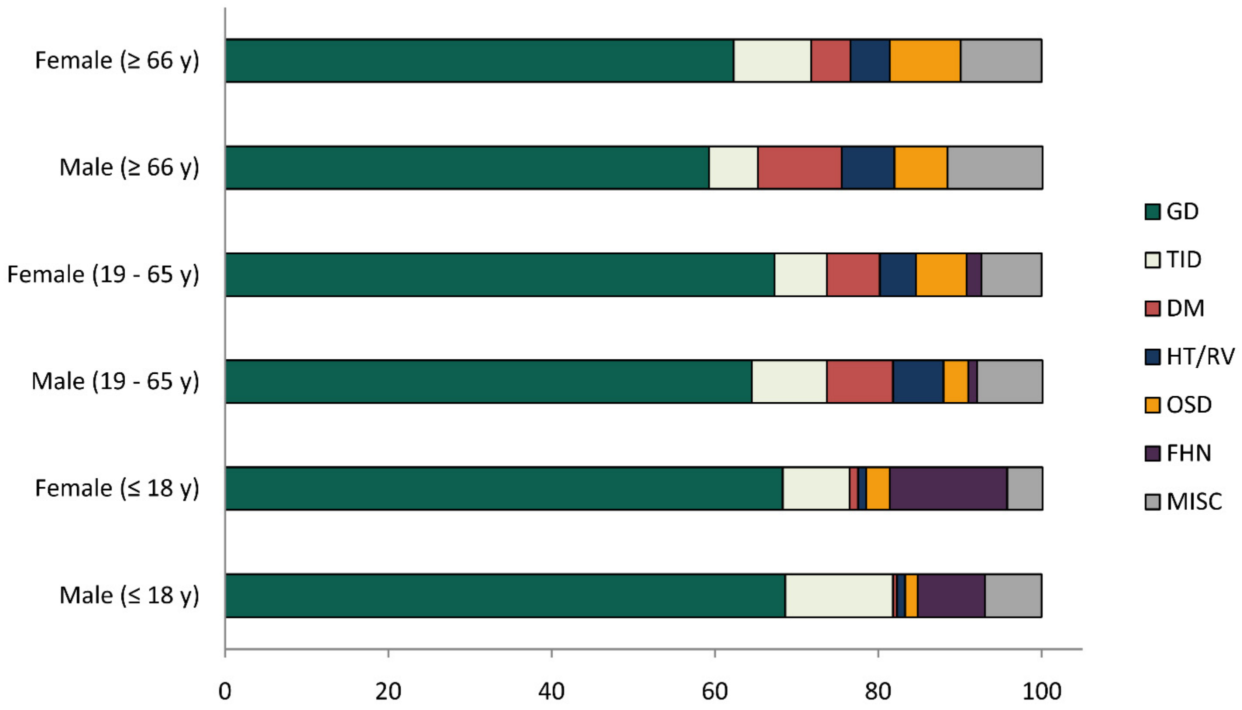

Suppl. Figure 1. The figure shows the fraction of total of main diagnoses according to genders within the age groups. They are presented as percentages (%). GD: glomerular diseases, TID: tubulointerstitial diseases, DM: diabetes mellitus, HT/RV: hypertension/renal vascular disease, OSD: other systemic disease affecting the kidney, FHN: familial/hereditary nephropathies, MISC: miscellaneous diseases, y: years.

Suppl. Table 3: Percentage of glomerular diseases according to genders within the age groups

Category	All								
	≤ 18 y		*P*	19 – 65 y		*P*	≥ 66 y		*P*
	M	F		M	F		M	F	
IgAN (%)	41.4	19.7	*<0.0001*	31.6	12.6	*<0.0001*	14.8	5.7	*0.015*
FSGS (%)	25.0	25.4	*NS*	16.5	18.1	*NS*	8.6	14.0	*NS*
MN (%)	1.4	4.2	*NS*	18.2	10.6	*0.001*	35.2	29.9	*NS*
MCD (%)	20.7	19.0	*NS*	10.9	13.0	*NS*	9.4	7.0	*NS*
SLE/LN (%)	1.4	14.8	*<0.0001*	6.7	30.0	*<0.0001*	-	4.5	*0.018*
MPA (%)	0.7	6.3	*0.019*	6.1	8.8	*NS*	14.8	26.8	*NS*
GPA (%)	1.4	2.1	*NS*	1.7	0.9	*NS*	3.9	1.3	*NS*
EGPA (%)	-	-	-	0.2	0.2	-	0.8	1.9	*NS*
MPGN (%)	3.6	4.2	*NS*	3.1	1.8	*NS*	1.6	3.2	*NS*
anti-GBM (%)	0.7	1.4	*NS*	0.8	0.4	*NS*	0.8	1.3	*NS*
Other GN (%)	3.6	2.8	*NS*	4.2	3.7	*NS*	10.2	4.5	*NS*
n	140	142	-	478	454	-	128	157	-

Suppl. Table 3. The table shows the fraction of total of glomerular diseases according to genders within the age groups. They are presented as percentages (%). P values show the result of the statistical analysis of the difference between the genders within the age groups. Chi-square and Fisher's exact test were used. IgAN: IgA nephropathy – histologically proven and Henoch-Schönlein purpura/nephritis, FSGS: Focal segmental

glomerulosclerosis, MN: Membranous nephropathy (primary and secondary), MCD: Minimal change disease, SLE/LN: Systemic lupus erythematosus/Lupus nephritis, MPA: Microscopic polyangiitis, GPA: Granulomatosis with polyangiitis, EGPA: Eosinophilic granulomatosis with polyangiitis, MPGN: Membranoproliferative glomerulonephritis, Other GN: Other glomerulonephritis, NS: nonsignificant, y: years.

Suppl. Figure 2: Percentage of glomerular diseases according to genders within the age groups

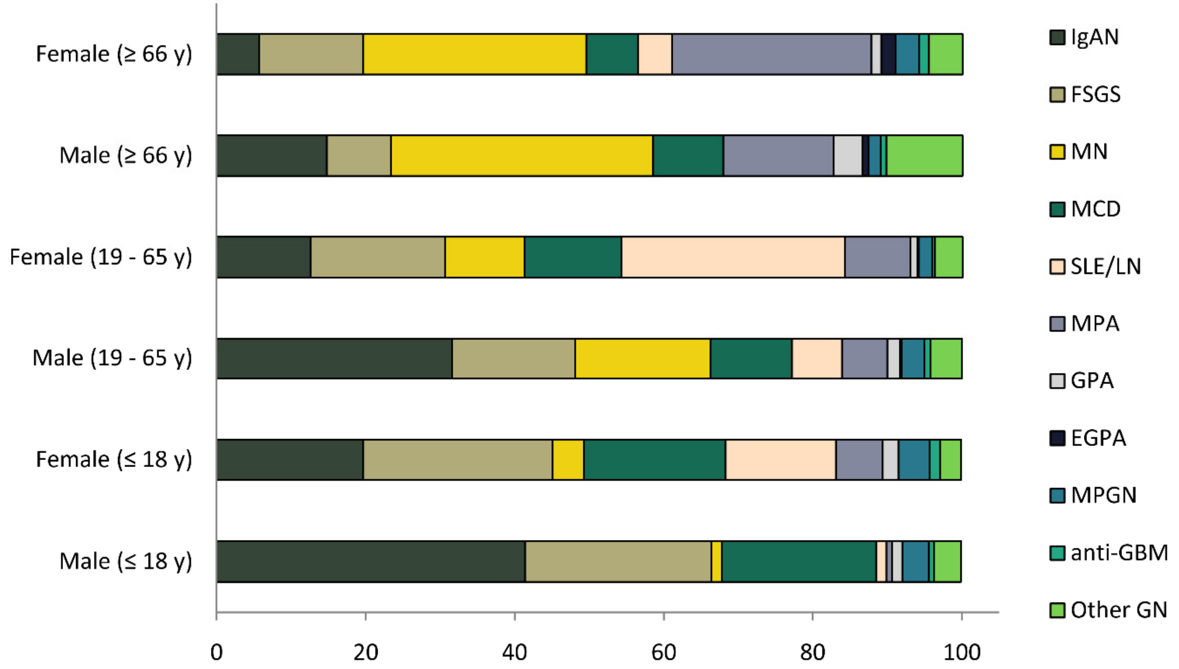

Suppl. Figure 2. The figure shows the fraction of total of glomerular diseases according to genders within the age groups. They are presented as percentages (%). IgAN: IgA nephropathy – histologically proven and Henoch-Schönlein purpura/nephritis, FSGS: Focal segmental glomerulosclerosis, MN: Membranous nephropathy (primary and secondary), MCD: Minimal change disease, SLE/LN: Systemic lupus erythematosus/Lupus nephritis, MPA: Microscopic polyangiitis, GPA: Granulomatosis with polyangiitis, EGPA: Eosinophilic granulomatosis with polyangiitis, MPGN: Membranoproliferative glomerulonephritis, Other GN: Other glomerulonephritis, y: years.
